# The effect of adjuvant radiotherapy after breast-conserving surgery in elderly women with T1-2N0 estrogen receptor-negative breast cancer

**DOI:** 10.1371/journal.pone.0288078

**Published:** 2023-08-03

**Authors:** Can Chen, Runlu Wang, Bing Wang, Yue Wu, Jingting Jiang

**Affiliations:** 1 Department of Oncology, The Third Affiliated Hospital of Soochow University, Changzhou, Jiangsu Province, China; 2 Respiratory Division, The Second Hospital of Hebei Medical University, Shijiazhuang, China; 3 Department of Rheumatology and Immunology, The Third Affiliated Hospital of Soochow University, Changzhou, Jiangsu Province, China; 4 Department of Tumor Biological Treatment, The Third Affiliated Hospital of Soochow University, Changzhou, Jiangsu Province, China; Local Health Authority Caserta: Azienda Sanitaria Locale Caserta, ITALY

## Abstract

**Purpose:**

To evaluate whether adjuvant radiotherapy (RT) following breast-conserving surgery (BCS) results in better survival among women ≥ 70 years with T1-2N0 estrogen receptor (ER)-negative breast cancer.

**Methods:**

In this retrospective cohort study, we included patients who met the inclusion criteria between 2010 and 2015 from the Surveillance, Epidemiology, and End Results (SEER) program. Univariate and Multivariate Cox proportional analysis were used to identify the risk factors for overall survival (OS) and breast cancer-specific survival (BCSS). Kaplan-Meier survival analysis was used to compare the prognosis of patients with or without adjuvant RT. Propensity score matching (PSM) was applied to perform a 1:1 matched case-control analysis.

**Results:**

A total of 4201 women were included in this study, with a median follow-up time of 64 months (range: 0–107 months). Of these patients, 2811 (66.9%) received adjuvant RT, while 1390 (33.1%) did not. Patients who did not receive adjuvant RT were more likely to be aged ≥ 80 years old, have a single marital status, larger tumors, and HER2-positive status (p < 0.05). Multivariate Cox proportional analysis indicated that receiving adjuvant RT was an independent factor associated with better OS and BCSS before and after PSM (P < 0.001). The survival curves before and after PSM showed that patients achieved an improved OS and BCSS from adjuvant RT (P < 0.005). In the subgroup analysis, there was no survival benefit trend from adjuvant RT in patients who were ≥ 80 years, or those with T1mic+T1a, T1b tumors.

**Conclusions:**

The use of RT following BCS in older women with T1-2N0 ER-negative breast cancer is associated with improve OS and BCSS. However, the potential benefit may be relatively limited for patients ≥ 80 years, or those with T1mic+T1a, T1b tumors.

## Introduction

Breast cancer has become the most common cancer worldwide, with nearly 2.3 million new cases in 2020 [1]. Adjuvant radiotherapy (RT) following Breast-conserving surgery (BCS) is the standard treatment for early stage breast cancer. Advanced age is a well-established prognostic factor associated with a decreased risk of tumor recurrence [2–4], and various randomized clinical trials (RCTs) have demonstrated slight improvement in local control of breast cancer in elderly patients, but no impact on distant metastasis-free or disease-specific survival has been observe [5–7], which suggests adjuvant RT may have a reduced benefit in elderly patients.

Several prospective studies showed a modest decrease in local recurrence when RT was added to elderly women with stage I ER-positive breast cancer receiving tamoxifen, but no difference was observed in terms of breast-cancer specific survival (BCSS) [6,8–12]. As a result, the National Comprehensive Cancer Network guidelines allow for the use of BCS plus endocrine therapy without adjuvant RT for this subset of patients [13]. However, it is currently not known whether omitting RT is also a safe alternative for elderly women with ER-negative breast cancer.

Our study aimed to evaluate the benefit of adjuvant RT after BCS among women ≥ 70 years with T1-2N0 ER-negative breast cancer.

## Materials and methods

### Patient selection

**Fig 1:**
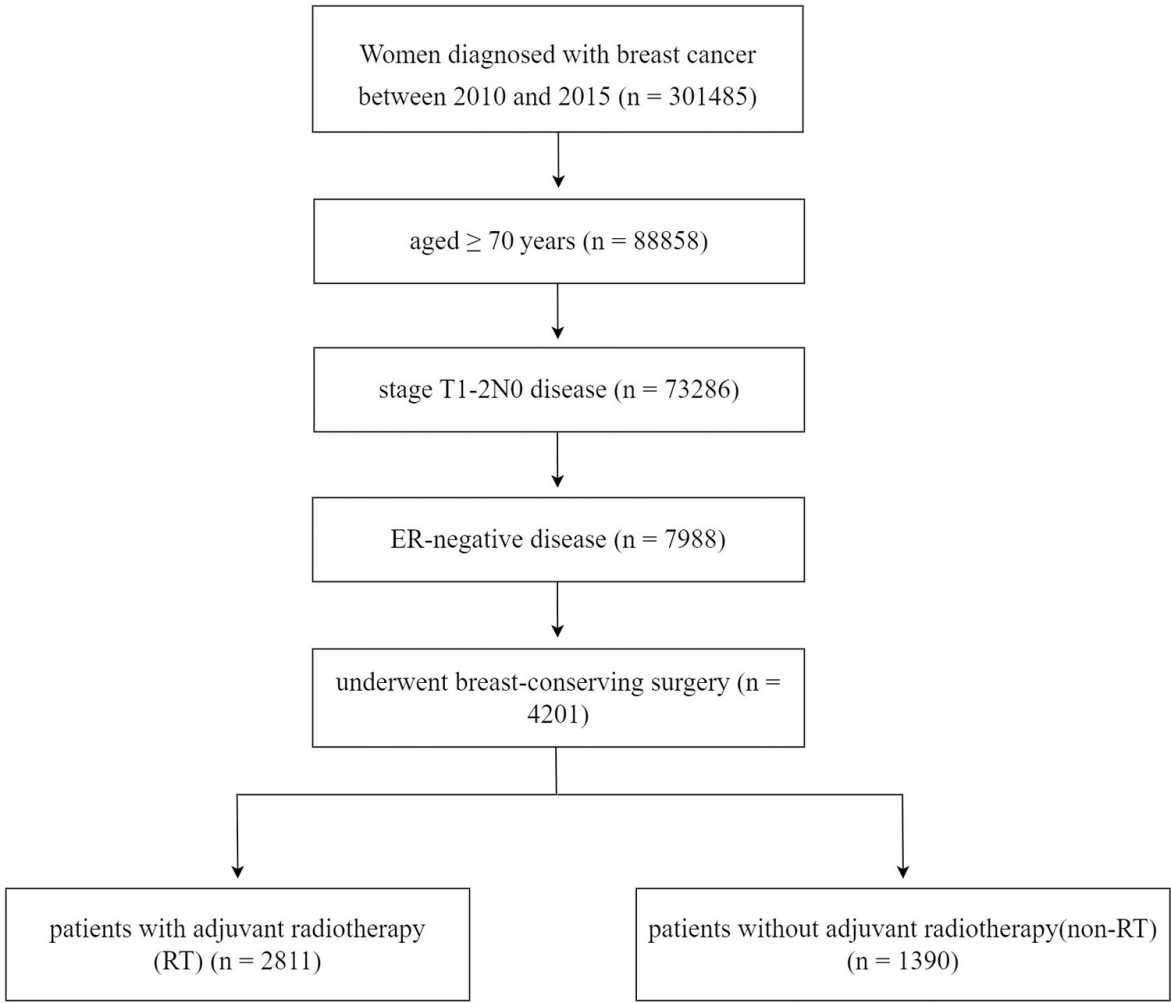
Flow chart of the inclusion and exclusion criteria.

The Surveillance, Epidemiology, and End Results (SEER) program is recognized as the authoritative source of cancer diagnosis, treatment, and survival data in the United States, covering approximately 28% of the population. We enrolled patients from the SEER program between 2010 and 2015 who had complete follow-up information and met the following criteria: 1) women diagnosed with breast cancer and aged ≥ 70 years; 2) stage T1-2N0 disease; 3) estrogen receptor (ER)-negative disease; 4) treated with or without adjuvant RT following BCS. Patients who were diagnosed with metastatic disease at breast cancer diagnosis, and who received systemic therapy before BCS were excluded (Fig 1). Notably, this study was considered exempt from the approval process of the Institutional Review Board as it used de-identified data from the SEER program.

### Measures

The following variables were included in this study: age at diagnosis, race/ethnicity, marital status, tumor Histology, grade, tumor (T) stage, progesterone receptor (PR), human epidermal growth factor receptor 2 (HER2) status, chemotherapy use, and adjuvant RT use. The primary endpoints were overall survival (OS) and breast cancer-specific survival (BCSS). OS was defined as the time from breast cancer diagnosis to death from any cause, and BCSS was defined as the time from the date of breast cancer diagnosis to the date of death from breast cancer.

### Statistical analysis

The chi-square test was used to compare the baseline characteristics of patients between the groups. Cox proportional hazard models were used to determine the prognostic factors for OS and BCSS. To reduce potential confounding in the retrospective studies, a 1:1 propensity score matching (PSM) method was used to create the matched cohorts. Kaplan-Meier survival analysis and log-rank tests were performed to compare the survival among different groups. All tests were two-sided, and P-value < 0.05 was applied to indicate statistical significance.

## Result

### Patient characteristics

**Fig 2:**
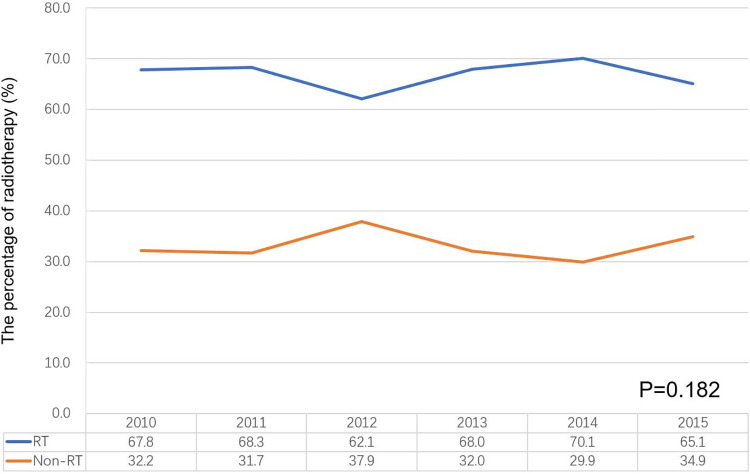
Change in the use of adjuvant radiotherapy following breast-conserving surgery between 2010 and 2015.

A total of 4201 patients were included in the final analysis with a median age of diagnosis of 75 years (range: 70–99 years). The majority of patients were White (n  =  3375, 80.3%), single marital status (n  =  2451, 58.3%), PR-negative (n  =  3947, 94.0%), HER2-negative (n  =  3347, 79.7%), and did not receive chemotherapy (n  =  2547, 60.6%). Of these patients, 2,811 (66.9%) received adjuvant RT, while 1390 (33.1%) patients did not. Notably, there were no significant differences in the percentage of patients receiving adjuvant RT over time (P = 0.182) (Fig 2). Demographic and clinical characteristics information of patients before and after PSM were shown in Table 1.

**Table 1:** Patient demographic and clinical characteristics information before and after PSM.

Characteristics	Before PSM				After PSM			
	Total (n = 4201)	RT (n = 2811)	Non-RT (n = 1390)	*P*-value	Total (n = 2714)	RT (n = 1357)	Non-RT (n = 1357)	*P*-value
**Age**				< 0.001				0.948
70–74	1714 (40.8%)	1260 (44.8%)	454 (32.7%)		913 (33.6%)	459 (33.8%)	454 (33.5%)	
75–79	1139 (27.1%)	812 (28.9%)	327 (23.5%)		647 (23.8%)	320 (23.6%)	327 (24.1%)	
≥ 80	1348 (32.1%)	739 (26.3%)	609 (43.8%)		1154 (42.5%)	578 (42.6%)	576 (42.4%)	
**Race**				0.727				0.618
White	3375 (80.3%)	2267 (80.6%)	1108 (79.7%)		2139 (78.8%)	1060 (78.1%)	1079 (79.5%)	
Black	595 (14.2%)	394 (14.0%)	201 (14.5%)		412 (15.2%)	215 (15.8%)	197 (14.5%)	
Other	231 (5.5%)	150 (5.3%)	81 (5.8%)		163 (6.0%)	82 (6.0%)	81 (6.0%)	
**Marital status**				< 0.001				0.811
Married	1750 (41.7%)	1262 (44.9%)	488 (35.1%)		981 (36.1%)	494 (36.4%)	487 (35.9%)	
Single	2451 (58.3%)	1549 (55.1%)	902 (64.9%)		1733 (63.9%)	863 (63.6%)	870 (64.1%)	
**Tumor Histology**				0.038				0.547
Ductal Carcinoma	3515 (83.7%)	2380 (84.7%)	1135 (81.7%)		2230 (82.2%)	1120 (82.5%)	1110 (81.8%)	
Lobular Carcinoma	64 (1.5%)	38 (1.4%)	26 (1.9%)		43 (1.6%)	18 (1.3%)	25 (1.8%)	
Other	622 (14.8%)	393 (14%)	229 (16.5%)		441 (16.2%)	219 (16.1%)	222 (16.4%)	
**Grade**				0.739				0.892
I	180 (4.3%)	123 (4.4%)	57 (4.1%)		107 (3.9%)	51 (3.8%)	56 (4.1%)	
II	1143 (27.2%)	777 (27.6%)	366 (26.3%)		695 (25.6%)	342 (25.2%)	353 (26.0%)	
III	2854 (67.9%)	1896 (67.4%)	958 (68.9%)		1895 (69.8%)	956 (70.4%)	939 (69.2%)	
IV	24 (0.6%)	15 (0.5%)	9 (0.6%)		17 (0.6%)	8 (0.6%)	9 (0.7%)	
**Laterality**				0.421				0.513
Left	2207 (52.5%)	1464 (52.1%)	743 (53.5%)		1452 (53.5%)	735 (54.2%)	717 (52.8%)	
Right	1994 (47.5%)	1347 (47.9%)	647 (46.5%)		1262 (46.5%)	622 (45.8%)	640 (47.2%)	
**T stage**				< 0.001				0.831
T1mic+T1a	406 (9.7%)	294 (10.5%)	112 (8.1%)		211 (7.8%)	99 (7.3%)	112 (8.3%)	
T1b	847 (20.2%)	619 (22%)	228 (16.4%)		457 (16.8%)	229 (16.9%)	228 (16.8%)	
T1c	1730 (41.2%)	1173 (41.7%)	557 (40.1%)		1120 (41.3%)	564 (41.6%)	556 (41.0%)	
T2	1218 (29.0%)	725 (25.8%)	493 (35.5%)		926 (34.1%)	465 (34.3%)	461 (34.0%)	
**PR status**				0.735				0.452
Negative	3947 (94.0%)	2644 (94.1%)	1303 (93.7%)		2562 (94.4%)	1286 (94.8%)	1276 (94.0%)	
Positive	254 (6.0%)	167 (5.9%)	87 (6.3%)		152 (5.6%)	71 (5.2%)	81 (6.0%)	
**HER2 status**				0.006				0.676
Negative	3347 (79.7%)	2274 (80.9%)	1073 (77.2%)		2120 (78.1%)	1065 (78.5%)	1055 (77.7%)	
Positive	854 (20.3%)	537 (19.1%)	317 (22.8%)		594 (21.9%)	292 (21.5%)	302 (22.3%)	
**Chemotherapy**				< 0.001				0.207
Not done	2547 (60.6%)	1593 (56.7%)	954 (68.6%)		1810 (66.7%)	889 (65.5%)	921 (67.9%)	
Done	1654 (39.4%)	1218 (43.3%)	436 (31.4%)		904 (33.3%)	468 (34.5%)	436 (32.1%)	

### Predictors associated with receiving adjuvant RT

The multivariate logistic regression analysis showed that age, marital status, T stage, and HER2 status were found to be strong predictors of radiation administration. Patients aged ≥ 80 years, with a single marital status, T2 stage disease, and HER2 positive status were less likely to receive postoperative RT (Table 2).

**Table 2:** Predictors of receipt of adjuvant radiotherapy using multivariable analysis.

Characteristic	OR	95%CI	*P*-value
**Age**			
70–74	Reference		
75–79	0.91	0.77–1.08	0.303
≥ 80	0.48	0.41–0.56	<0.001
**Race**			
White	Reference		
Black	0.93	0.77–1.13	0.482
Other	0.88	0.67–1.18	0.400
**Marital status**			
Married	Reference		
Single	0.79	0.68–0.91	0.001
**Tumor Histology**			
Ductal Carcinoma	Reference		
Lobular Carcinoma	0.72	0.43–1.23	0.225
Other	0.83	0.69–1.00	0.047
**Grade**			
I	Reference		
II	1.00	0.70–1.40	0.981
III	0.95	0.67–1.33	0.782
IV	1.00	0.41–2.55	0.994
**Laterality**			
Left	Reference		
Right	1.06	0.93–1.21	0.369
**T stage**			
T1mic+T1a	Reference		
T1b	1.03	0.79–1.35	0.823
T1c	0.83	0.65–1.06	0.133
T2	0.62	0.48–0.80	<0.001
**PR status**			
Negative	Reference		
Positive	0.98	0.75–1.30	0.890
**HER2 status**			
Negative	Reference		
Positive	0.77	0.66–0.91	0.002

### Survival analyses in the whole SEER cohort

**Fig 3:**
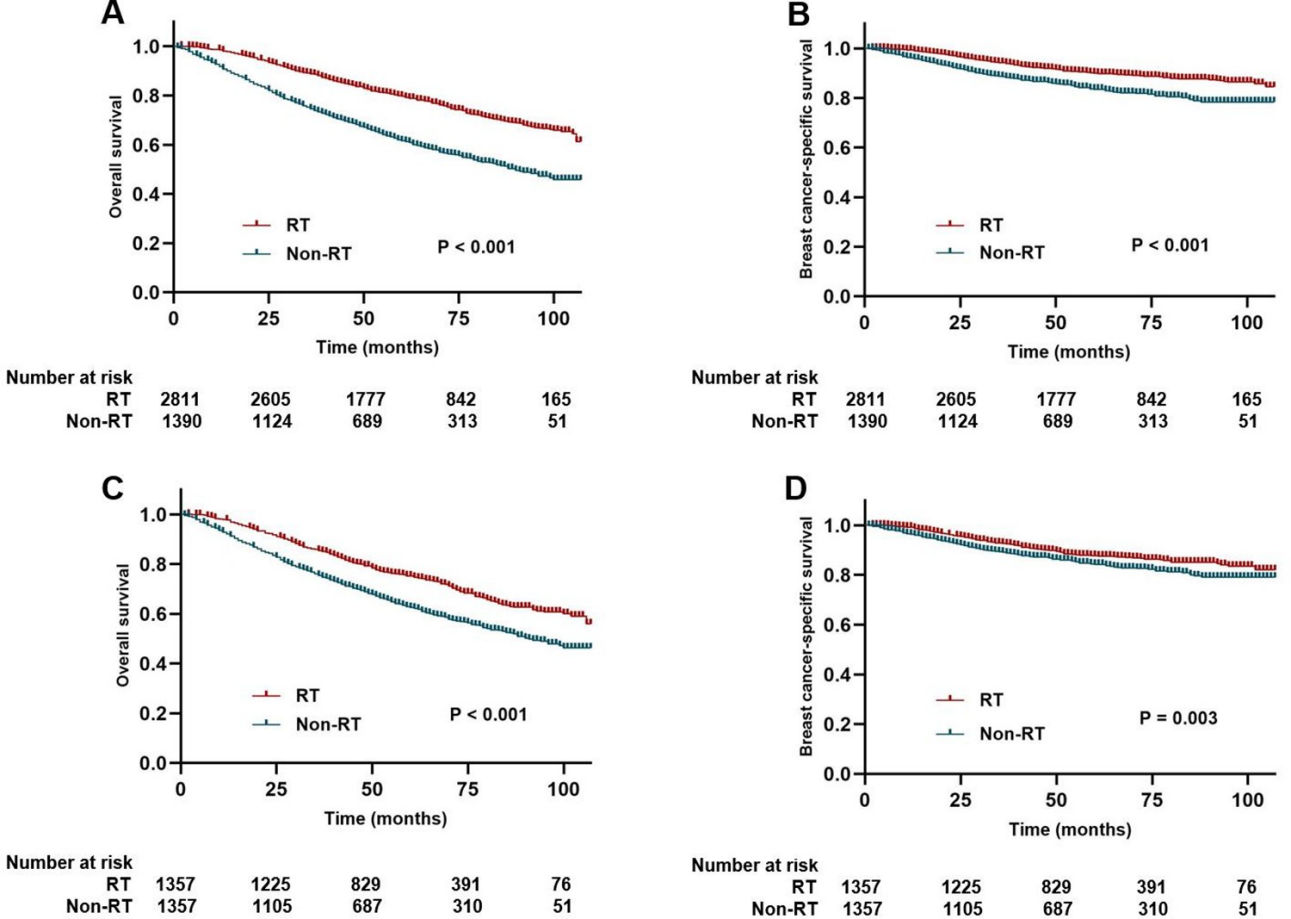
Kaplan–Meier curves for all patients with and without adjuvant radiotherapy. (A) OS curve of the non-RT group versus RT group before PSM; (B) BCSS curve of the non-RT group versus RT group before PSM; (C) OS curve of the non-RT group versus RT group after PSM; (D) BCSS curve of the non-RT group versus RT group after PSM.

During a median follow-up period of 64 months (range: 0–107 months) in the whole SEER cohort, 1233 (29.4%) patients died, with 487 (39.5%) of those deaths attributed to breast cancer. At 5 years, the OS and BCSS for the entire cohort were 73.7% and 88.0%, respectively, and at 8 years, these rates decreased to 60.4% and 83.9%, respectively. All baseline characteristics were analyzed through univariable and multivariate analysis to assess the impact on OS and BCSS. Univariate analysis showed that older age (≥ 80 years), single marital status, poorer tumor differentiation grade (III and IV), and higher T stage (T1c and T2) were associated with worse OS and BCSS, while adjuvant RT was strongly associated with improved survival (OS: HR 0.48, 95% CI 0.43–0.54, p < 0.001; BCSS: HR 0.56, 95% CI 0.47–0.67, p < 0.001). Multivariate analysis revealed that adjuvant RT was an independent prognostic factor for both OS and BCSS (p < 0.001) (Table 3). Furthermore, Kaplan-Meier curve analysis demonstrated that patients who received adjuvant RT had better OS (P < 0.001) and BCSS (P < 0.001) compared to those who did not (Fig 3A and 3B).

**Table 3:** Univariate and multivariate analyses of OS and BCSS before PSM.

Characteristics	Overall survival				Breast cancer-specific survival			
	Univariate analysis		Multivariate analysis		Univariate analysis		Multivariate analysis	
	HR (95% CI)	*P*-value	HR (95% CI)	*P*-value	HR (95% CI)	*P*-value	HR (95% CI)	*P*-value
**Age**								
70–74	Reference		Reference		Reference		Reference	
75–79	1.38 (1.18–1.62)	<0.001	1.24 (1.05–1.46)	0.010	1.24 (0.98–1.58)	0.079	1.19 (0.94–1.52)	0.155
≥ 80	3.08 (2.69–3.52)	<0.001	2.04 (1.76–2.37)	<0.001	2.21 (1.79–2.72)	<0.001	1.69 (1.34–2.14)	<0.001
**Race**								
White	Reference		Reference		Reference		Reference	
Black	1.06 (0.90–1.24)	0.476	1.06 (0.90–1.25)	0.467	1.17 (0.91–1.49)	0.216	1.14 (0.89–1.46)	0.289
Other	0.67 (0.50–0.90)	0.007	0.65 (0.49–0.87)	0.004	0.62 (0.38–1.00)	0.052	0.63 (0.39–1.02)	0.059
**Marital status**								
Married	Reference		Reference		Reference		Reference	
Single	1.58 (1.40–1.78)	<0.001	1.17 (1.03–1.32)	0.013	1.29 (1.07–1.55)	0.007	1.00 (0.82–1.21)	0.961
**Tumor Histology**								
Ductal Carcinoma	Reference		Reference		Reference		Reference	
Lobular Carcinoma	1.03 (0.66–1.60)	0.904	1.01 (0.65–1.58)	0.966	0.26 (0.06–1.04)	0.057	0.29 (0.07–1.17)	0.083
Other	1.00 (0.86–1.18)	0.959	0.96 (0.81–1.12)	0.586	0.92 (0.71–1.19)	0.509	0.87 (0.67–1.14)	0.321
**Grade**								
I	Reference		Reference		Reference		Reference	
II	1.07 (0.77–1.48)	0.683	1.09 (0.78–1.51)	0.611	1.25 (0.67–2.34)	0.487	1.17 (0.62–2.20)	0.623
III	1.48 (1.09–2.02)	0.012	1.44 (1.05–1.98)	0.025	2.38 (1.31–4.33)	0.005	1.78 (0.96–3.28)	0.066
IV	2.76 (1.45–5.24)	0.002	1.91 (1.00–3.65)	0.050	6.11 (2.37–15.76)	<0.001	3.42 (1.31–8.91)	0.012
**Laterality**								
Left	Reference		Reference		Reference		Reference	
Right	0.96 (0.86–1.07)	0.468	0.98 (0.87–1.09)	0.691	0.99 (0.83–1.18)	0.902	1.01 (0.85–1.21)	0.887
**T stage**								
T1mic+T1a	Reference		Reference		Reference		Reference	
T1b	1.25 (0.95–1.65)	0.116	1.28 (0.97–1.69)	0.087	1.68 (0.93–3.05)	0.086	1.64 (0.91–2.99)	0.102
T1c	1.81 (1.40–2.33)	<0.001	1.90 (1.46–2.45)	<0.001	3.14 (1.82–5.41)	<0.001	3.08 (1.78–5.34)	<0.001
T2	3.03 (2.35–3.90)	<0.001	2.96 (2.27–3.84)	<0.001	7.18 (4.19–12.30)	<0.001	6.37 (3.67–11.05)	<0.001
**PR status**								
Negative	Reference		Reference		Reference		Reference	
Positive	0.81 (0.65–1.00)	0.055	0.88 (0.70–1.09)	0.248	0.66 (0.48–0.91)	0.011	0.70 (0.51–0.97)	0.031
**HER2 status**								
Negative	Reference		Reference		Reference		Reference	
Positive	1.13 (0.98–1.31)	0.095	1.06 (0.92–1.23)	0.427	1.21 (0.96–1.53)	0.104	1.21 (0.95–1.53)	0.118
**Radiotherapy**								
Not done	Reference		Reference		Reference		Reference	
Done	0.48 (0.43–0.54)	<0.001	0.62 (0.55–0.70)	<0.001	0.56 (0.47–0.67)	<0.001	0.70 (0.58–0.84)	<0.001
**Chemotherapy**								
Not done	Reference		Reference		Reference		Reference	
Done	0.47 (0.41–0.53)	<0.001	0.52 (0.45–0.60)	<0.001	0.78 (0.64–0.94)	0.008	0.72 (0.58–0.89)	0.003

### Survival analysis in propensity score-matched cohort

In the matched cohort, univariate analysis revealed similar prognostic factors for OS and BCSS as the unmatched cohort, including age, marital status, Grade, T stage, chemotherapy, and radiotherapy (Table 4). The multivariate analysis indicated that adjuvant RT was an independent prognostic factor for both OS and BCSS; the OS (HR 0.62, 95% CI 0.54–0.70, p < 0.001) and BCSS (HR 0.71, 95% CI 0.58–0.88, p = 0.002) of patients who received postoperative RT were better than those who did not (Table 4). Additionally, age, T stage, and chemotherapy were independent indicators for both OS and BCSS. Furthermore, patients who received postoperative RT experienced a significant improvement in OS (5-year OS: 75.2% vs. 63.2%; 8-year OS: 60.7% vs. 47.8%, p < 0.001) and BCSS (5-year BCSS: 87.7% vs. 84.2%; 8-year BCSS: 83.4% vs. 79.0%, p = 0.003) compared to those who did not (Fig 3C and 3D).

**Table 4:** Univariate and multivariate analyses of OS and BCSS after PSM.

Characteristics	Overall survival				Breast cancer-specific survival			
	Univariate analysis		Multivariate analysis		Univariate analysis		Multivariate analysis	
	HR (95% CI)	*P*-value	HR (95% CI)	*P*-value	HR (95% CI)	*P*-value	HR (95% CI)	*P*-value
**Age**								
70–74	Reference		Reference		Reference		Reference	
75–79	1.41 (1.15–1.73)	0.001	1.28 (1.04–1.57)	0.019	1.48 (1.09–2.00)	0.012	1.44 (1.06–1.96)	0.020
≥ 80	2.78 (2.36–3.28)	<0.001	2.06 (1.72–2.47)	<0.001	2.16 (1.67–2.80)	<0.001	1.91 (1.43–2.54)	<0.001
**Race**								
White	Reference		Reference		Reference		Reference	
Black	1.00 (0.84–1.20)	0.971	1.07 (0.89–1.28)	0.497	1.18 (0.89–1.55)	0.249	1.22 (0.92–1.62)	0.160
Other	0.78 (0.58–1.05)	0.104	0.74 (0.55–1.00)	0.049	0.75 (0.45–1.24)	0.257	0.72 (0.44–1.20)	0.210
**Marital status**								
Married	Reference		Reference		Reference		Reference	
Single	1.50 (1.31–1.73)	<0.001	1.18 (1.02–1.37)	0.024	1.28 (1.03–1.60)	0.028	1.04 (0.83–1.31)	0.731
**Tumor Histology**								
Ductal Carcinoma	Reference		Reference		Reference		Reference	
Lobular Carcinoma	0.86 (0.50–1.45)	0.565	0.89 (0.52–1.53)	0.680	0.16 (0.02–1.16)	0.070	0.20 (0.03–1.42)	0.108
Other	1.04 (0.88–1.24)	0.625	1.04 (0.87–1.24)	0.683	1.00 (0.76–1.33)	0.998	1.00 (0.75–1.34)	0.999
**Grade**								
I	Reference		Reference		Reference		Reference	
II	0.95 (0.65–1.39)	0.799	1.10 (0.75–1.61)	0.639	1.14 (0.54–2.38)	0.731	1.26 (0.60–2.65)	0.539
III	1.36 (0.95–1.95)	0.091	1.47 (1.01–2.13)	0.042	2.14 (1.06–4.32)	0.034	1.88 (0.91–3.85)	0.086
IV	2.62 (1.29–5.35)	0.008	1.86 (0.90–3.82)	0.092	5.13 (1.68–15.68)	0.004	3.04 (0.98–9.42)	0.053
**Laterality**								
Left	Reference		Reference		Reference		Reference	
Right	0.99 (0.87–1.12)	0.849	0.97 (0.86–1.11)	0.684	1.03 (0.84–1.27)	0.746	1.02 (0.83–1.25,)	0.867
**T stage**								
T1mic+T1a	Reference		Reference		Reference		Reference	
T1b	1.24 (0.87–1.75)	0.231	1.30 (0.92–1.84)	0.142	2.07 (0.91–4.70)	0.082	2.05 (0.90–4.66)	0.088
T1c	1.67 (1.22–2.28)	0.001	1.81 (1.32–2.49)	<0.001	3.52 (1.65–7.55)	0.001	3.51 (1.63–7.55)	0.001
T2	2.68 (1.97–3.66)	<0.001	2.89 (2.10–3.98)	<0.001	7.85 (3.70–16.69)	<0.001	7.38 (3.44–15.83)	<0.001
**PR status**								
Negative	Reference		Reference		Reference		Reference	
Positive	0.95 (0.72–1.25)	0.724	1.07 (0.81–1.41)	0.621	1.24 (0.75–2.05)	0.398	1.24 (0.75–2.05)	0.398
**HER2 status**								
Negative	Reference		Reference		Reference		Reference	
Positive	1.27 (1.08–1.50)	0.004	1.27 (1.08–1.50)	0.004	1.33 (1.02–1.74)	0.038	1.23 (0.94–1.63)	0.136
**Radiotherapy**								
Not done	Reference		Reference		Reference		Reference	
Done	0.63 (0.55–0.72)	<0.001	0.62 (0.54–0.70)	<0.001	0.73 (0.59–0.89)	0.003	0.71 (0.58–0.88)	0.002
**Chemotherapy**								
Not done	Reference		Reference		Reference		Reference	
Done	0.42 (0.36–0.49)	<0.001	0.48 (0.40–0.58)	<0.001	0.72 (0.58–0.91)	0.006	0.74 (0.57–0.95)	0.020

### Survival analysis stratified by clinical characteristics

**Fig 4:**
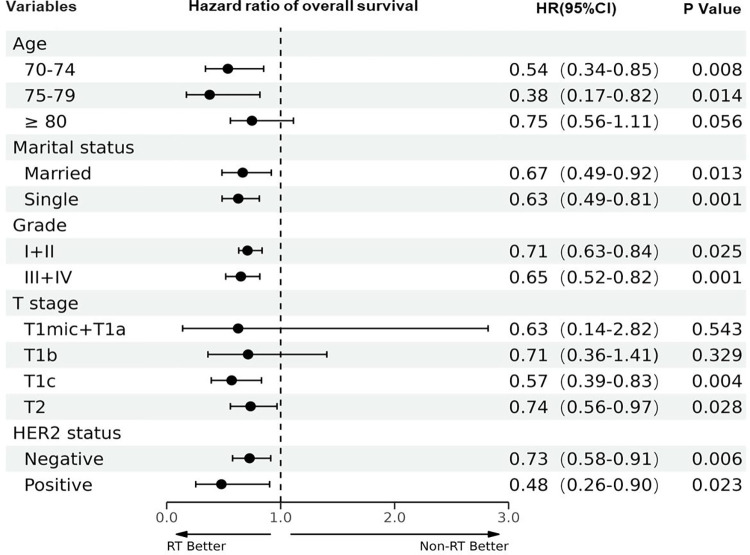
The forest plots of sub-group analysis.

In order to identify a specific subgroup of breast cancer patients who may benefit from adjuvant RT, we conducted an exploratory subgroup analysis in the propensity score matched cohort. Our findings suggest that patients respond differently to adjuvant RT depending on several factors. Specifically, the administration of adjuvant RT was significantly associated with prolonged OS in patients with a younger age (< 80 years), and those with a higher T stage. Moreover, adjuvant RT is associated with improved OS regardless of tumor grade, marital status, and HER2 status. These observations suggest that postoperative RT may serve as an effective treatment option for breast cancer patients with specific clinical and pathological characteristics. To visualize the relationship between each patient characteristic and the receipt of adjuvant RT, we plotted the hazard ratio and 95% confidence interval for OS in Fig 4.

## Discussion

The benefits of adjuvant RT for elderly women with early stage ER-negative breast cancer remain uncertain due to the limited research conducted in this population. In a meta-analysis of 17 randomized trials investigating adjuvant RT after BCS, only 23 women aged ≥ 70 years and had T1-T2N0 ER-negative breast cancer were included. This particular group of women represented less than 1% of the total population studied. The study demonstrated that patients with ER-negative breast cancer have an increased risk of tumor recurrence [7]. According to Other studies, both prospective and retrospective, it seems likely that patients with ER-negative tumors may not receive the same advantages from adjuvant RT as those with ER-positive tumors [14]. Therefore, it is possible that the increased danger of tumor recurrence in patients with ER-negative tumors may not necessarily translate into heightened benefits from adjuvant RT. In the era of precise medicine and personalized treatment, it is extremely important to understand the prognostic value of adjuvant RT particularly for elderly women who have ER-negative tumors.

In our study, the proportion of patients aged ≥ 70 years with T1-T2N0 ER-negative breast cancer who received RT after BCS was not significantly different from 2010 to 2015 (P = 0.182). Moreover, we found that age, marital status, tumor grade, and HER2 status were the main factors associated with RT decision, which is consistent with previous clinical practice [15,16]. Numerous studies have demonstrated that older breast cancer patients with ER-negative tumors have higher rates of recurrence and breast cancer-specific mortality [17–19]. Furthermore, within the cohort of patients with ER-negative and PR-negative breast cancer, older individuals are more prone to die from the disease than their younger counterparts [20,21]. Although radiotherapy may lead to acute and chronic treatment-related toxicity, as well as increased medical costs [22,23], researchers have discovered that its use does not have a negative impact on the quality of life of elderly patients [5,24,25]. A study conducted by Weiss et al. based on population analysis revealed that older patients with ER-negative breast cancer had a higher breast cancer-specific mortality rate and lower utilization of radiotherapy. Their findings indicate that ER-negative breast cancer should be considered a distinct entity from ER-positive breast cancer and should be treated with more aggressive methods [20]. In our study, we found that the use of adjuvant radiation after BCS in older women with T1-2N0 ER-negative breast cancer is associated with improve OS and BCSS before and after PSM.

It is still uncertain whether a specific group of elderly women with early-stage ER-negative tumors may not need adjuvant RT. Our findings have demonstrated an improve OS among all subgroups of patients according to Marital status or Grade or HER2 status after BCS with RT; However, when analyzing patients aged ≥ 80 years, or those with T1mic+T1a, T1b tumors, the difference in OS with and without RT is no longer statistically significant. In addition, previous studies have shown that radiotherapy does not significantly reduce the risk of local regional recurrence in older age [2–4] or earlier T stage [7,26,27]. These studies will help guide management decisions for women ≥ 70 years with T1-2N0 ER-negative breast cancer.

Our study used a population-based cancer registry; unlike single-institution studies, which inevitably have a referral bias, the SEER database provides a more realistic clinical practice environment with information from all levels of healthcare institutions. Although there are many strengths of this study including the large sample size, PSM test and subgroup analysis, we acknowledge some limitations to our study. First, there were no information regarding postoperative RT in the SEER database, including clinical target volume and radiation regimen, which may cause confusion. Second, data on chemotherapy, such as regimen and courses, were also unavailable, so that further case-control studies failed to be performed. Finally, the SEER database did not include local recurrence and disease-free survival, which made the local control benefit of radiation therapy unanalyzable.

## Conclusions

The use of adjuvant RT is associated with improve OS and BCSS among elderly women ≥ 70 years with T1-2N0 ER-negative breast cancer. However, the potential benefit may be relatively limited in the subgroups of women ≥ 80 or with T1mic+T1a, T1b tumors.
